# Drain output volume after pancreaticoduodenectomy is a useful warning sign for postoperative complications

**DOI:** 10.1186/s12893-021-01285-1

**Published:** 2021-06-03

**Authors:** Taro Fukui, Hiroshi Noda, Fumiaki Watanabe, Takaharu Kato, Yuhei Endo, Hidetoshi Aizawa, Nao Kakizawa, Masahiro Iseki, Toshiki Rikiyama

**Affiliations:** Department of Surgery, Saitama Medical Center, Jichi Medical University, 1-847 Amanuma-cho, Omiya-ku, Saitama, 330-8503 Japan

**Keywords:** Drain output, Pancreaticoduodenectomy, Pancreatic fistula, Chyle leak

## Abstract

**Introduction:**

The drain output volume (DOV) after pancreaticoduodenectomy (PD) is an easily assessable indicator in clinical settings. We explored the utility of the DOV as a possible warning sign of complications after PD.

**Methods:**

A total of 404 patients undergoing PD were considered for inclusion. The predictability of the DOV for overall morbidity, major complications, intraabdominal infection (IAI), clinically relevant (CR) postoperative pancreatic fistula (POPF), CR delayed gastric emptying (DGE), CR chyle leak (CL), and CR post-pancreatectomy hemorrhaging (PPH) was evaluated.

**Results:**

One hundred (24.8%) patients developed major complications, and 131 (32.4%) developed IAI. Regarding CR post-pancreatectomy complications, 75 (18.6%) patients developed CR-POPF, 23 (5.7%) developed CR-DGE, 20 (5.0%) developed CR-CL, and 28 (6.9%) developed CR-PPH. The median DOV on postoperative day (POD) 1 and POD 3 was 266 and 234.5 ml, respectively. A low DOV on POD 1 was an independent predictor of CR-POPF, and a high DOV on POD 3 was an independent predictor of CR-CL. A receiver operating characteristics (ROC) analysis revealed that the DOV on POD 1 had a negative predictive value (area under the curve [AUC] 0.655, sensitivity 65.0%, specificity 65.3%, 95% confidence interval [CI]: 0.587–0.724), with a calculated optimal cut-off value of 227 ml. An ROC analysis also revealed that the DOV on POD 3 had a positive predictive value (AUC 0.753, sensitivity 70.1%, specificity 75.0%, 95% CI: 0.651–0.856), with a calculated optimal cut-off value of 332 ml.

**Conclusion:**

A low DOV on POD 1 might be a postoperative warning sign for CR-POPF, similar to high drain amylase (DA) on POD 1, high DA on POD 3, and high CRP on POD 3. When the DOV on POD 1 after PD was low, surgeons should evaluate the reasons of a low DOV. A high DOV on POD 3 was a postoperative warning sign CR-CL, and might require an appropriate management of protein loss.

## Introduction

While the actual need for drain placement after pancreaticoduodenectomy (PD) is still a matter of some debate, it remains standard practice [1, 2]. The drain output after PD is easily assessable after PD, and among measurements of the drain output, the International Study Group of Pancreatic Surgery (ISGPS) proposed the definition of postoperative pancreatic fistula (POPF) using drain amylase (DA) on postoperative day (POD)3 [3]. DA has been used in clinical settings to predict POPF, and a high DA on POD 1 and POD 3 has been repeatedly reported to be a strong postoperative warning sign of clinically relevant (CR)-POPF [1, 4–7]. In addition, recent studies have shown that a positive bacterial culture in drain output after PD is associated with increased incidences of complications, including CR-POPF, major complications and reoperation [8, 9]. Thus, the drain output after PD has been studied for its predictability for postoperative complications.

The drain output volume (DOV) after PD is also an easily assessable indicator in the clinical setting. The value ranges widely among cases, and a high DOV after PD is frequently encountered. However, to our knowledge, no reports have comprehensively described the relationship among the DOV after PD and overall complications, major complications and post-pancreatectomy complications, which include POPF [3], delayed gastric emptying (DGE) [10], chyle leak (CL) [11] and post-pancreatectomy hemorrhage (PPH) [12]. Only a few studies have reported that a high DOV is associated with severe dehydration and protein loss, and patients with a high DOV may require a longer hospital stay to manage their massive fluid loss [13, 14].

In this retrospective study, we evaluated the impact of the DOV on the postoperative morbidity after PD. In particular, we focused the relationships between the DOV and CR post-pancreatectomy complications. We sought to clarify the utility of the DOV as a possible postoperative warning signs of complications after PD.

## Material and methods

### Patients

This study was reviewed and approved by the Ethics Committee of Jichi Medical University, and the Ethics Committee of Jichi Medical University waived the need for informed consent because of the study’s retrospective nature. A total of 404 patients undergoing PD in our hospital between January 2006 and April 2020 were considered for the study. Ten patients who underwent combined with hemihepatectomy or more and three patients who underwent urgent PD were excluded.

### Surgical procedure and perioperative management

PD or subtotal stomach-preserving PD was performed as previously reported [15]. In the majority of malignant cases, lymph node (LN) dissection was carried out along the hepatico-duodenal ligament, common hepatic artery and superior mesenteric vein as well as along the right side of the superior mesenteric artery. In all cases, the methods of reconstruction were as follows: a jejunal loop was lifted, and pancreaticojejunostomy was performed by duct-to-mucosa anastomosis with external drainage. The stump of the pancreas was tightly affixed to the jejuna serosa by interrupted sutures. Hepaticojejunostomy was then performed by interrupted sutures with external drainage. At the end of the procedure, two closed continuous suction drainage tube were placed close to the pancreatic and biliary anastomoses and pulled through the right flank.

Postoperatively, the assessment of the DA was routinely performed on POD 1 and POD 3. The drain output from two closed continuous suction drainage tubes was collected in dedicated bags. When we discarded the drain output, the volumes were measured at all times. The daily DOV was a sum of drain output during 24 h, and it was recorded in the medical chart until drain removal. The white blood cell (WBC) count and C-reactive protein (CRP) level were measured on POD 1 and POD 3. The drains were removed progressively from POD 4, but drainage durations were left to the discretion of the surgeon, depending on the DA, volume and characteristics of the drain output, and signs of inflammations (e.g. WBC count and CRP). Oral intake was started from POD 4–5. Feeding tubes and total parental nutrition were not used routinely in the postoperative course.

### Data acquisition

The preoperative and postoperative data were collected by a review of the patients’ medical records. The preoperative variables included the age, gender, pathological diagnosis, preoperative body mass index (BMI), preoperative biliary drainage, preoperative hemoglobin, preoperative serum total protein, preoperative serum albumin and preoperative general comorbidities, including diabetes mellitus, hypertension, ischemic heart disease, and liver cirrhosis. The intraoperative variables also included operative time, estimated blood loss, the need for blood transfusion, portal vein or superior mesenteric vein resection, additional organ resection and manipulation of para-aortic LNs. The pathological diagnosis was divided into pancreatic cancer and not pancreatic cancer, as PD for pancreatic cancer sometimes requires extensive LN dissection and superior mesenteric plexus dissection for R0 resection, which can lead to a high DOV after PD [14].

### Definitions of postoperative complications and grading of CR post-pancreatectomy complications.

Overall morbidity was defined as any complication. The severity of complications was graded per the Clavien–Dindo classification, and major complications were defined as any complication of grade ≥ 3 severity [16]. Intraabdominal infection (IAI) was defined as intraabdominal fluid collection with a positive culture or organ/space surgical site infection in the abdominal cavity [17]. The grade of post-pancreatectomy complications, including POPF, DGE, CL and PPH, was decided according to each criterion of the ISGPS [3, 10–12]. For each post-pancreatectomy complication, Grades B and C were considered CR, and these grades were assigned to any instance of CR-POPF, CR-DGE, CR-CL and CR-PPH [3, 10–12].

### Statistical analyses

Data are expressed as the number (percentage) or median (range). The Mann–Whitney U test was used to assess non-normally distributed data. Factors with a p value of < 0.05 in a univariate analysis were entered into a multivariable logistic regression analysis. The results of the multivariate analysis were expressed as the odds ratio (OR) and 95% confidence interval (CI). Receiver operative characteristic (ROC) curves for the DOV, DA, WBC counts and CRP on POD 1 and POD 3 were used to determine the cut-off value to predict the overall morbidity, major complications, IAI, CR-POPF, CR-DGE, CR-CL and CR-PPH. P values of < 0.05 were considered to indicate statistical significance. The correlation of the DOV on POD 3 with the serum total protein on POD 3, and serum albumin on POD 3 were assessed using the Spearman’s rank correlation coefficient analysis. All statistical analyses were conducted using the EZR software program [18].

## Results

### Patients’ characteristics

The demographic and perioperative data of the 404 patients are summarized in Table 1. The median DOV on POD 1 and POD 3 were 266 and 234.5 ml, respectively. The overall mortality and morbidity rates were 1.0% (n = 4) and 37.2% (n = 152), respectively. A total of 100 (24.8%) patients developed major complications, and 131 (32.4%) developed IAI. Regarding CR post-pancreatectomy complications, 75 (18.6%) patients developed CR-POPF, 23 (5.7%) developed CR-DGE, 20 (5.0%) developed CR-CL, and 28 (6.9%) developed CR-PPH. There were no patients who developed both CR-POPF and CR-CL.

**Table 1:** The demographic and perioperative variables of the 404 patients

Variables	
*Preoperative variables*	
Age (years)	70 (24–90)
Gender, male	251 (62.1%)
Diseases, pancreatic cancer	149 (36.9%)
Preoperative body mass index (kg/m^2^)	21.3 (14.0–35.2)
Preoperative biliary drainage, yes	227 (56.2%)
Preoperative hemoglobin (g/dl)	12.3 (7.0–16.5)
Preoperative total protein (g/dl)	6.9 (4.8–8.4)
Preoperative albumin (g/dl)	3.8 (1.7–5.0)
Diabetes mellitus, yes	102 (25.2%)
Hypertension, yes	137 (33.9%)
Ischemic heart disease, yes	23 (5.7%)
Liver cirrhosis, yes	1 (0.2%)
*Intraoperative and pathological variables*	
Operative time (min)	428 (214–940)
Estimated blood loss (ml)	700 (20–4200)
Blood transfusion, present	116 (28.7%)
Portal vein or superior mesenteric vein resection, yes	80 (19.8%)
Associated visceral resection, yes	21 (5.2%)
Paraaortic lymph node sampling, yes	204 (50.5%)
Number of lymph node harvested	22 (0–57)
Lymph node metastasis, yes	180 (44.6%)
*Postoperative variables*	
White blood cell count on POD1 (/μl)	10,395 (635–113,800)
C-reactive protein on POD1 (mg/dl)	9.50 (0.09–25.84)
Drain output volume on POD1 (ml)	266(15–1490)
Drain amylase on POD1 (U/l)	716 (0–95,100)
White blood cell count on POD3 (/μl)	8580 (730–26,230)
C-reactive protein on POD3 (mg/dl)	12.12 (0.87–42.32)
Drain output volume on POD3 (ml)	234.5 (0–1573)
Drain amylase on POD3 (U/l)	102.5 (3–17,200)

*POD* postoperative day

### DOV and postoperative complications

**Fig. 1:**
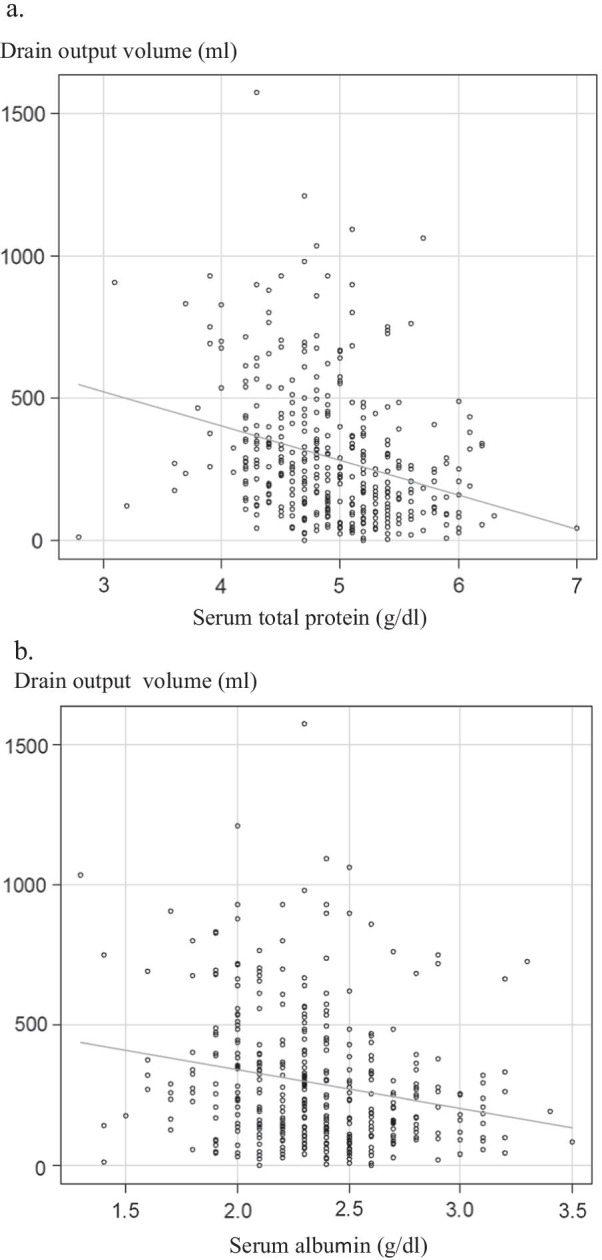
Results of a Spearman’s rank correlation coefficient analysis to assess the correlation between the DOV on POD 3 and the serum total protein on POD 3 (**a**) and serum albumin on POD 3 (**b**)

Table 2 shows the correlations among the DOV on POD 1 or POD 3 and the rates of overall morbidity (Table 2a), major complications (Table 2b), IAI (Table 2c), CR-POPF (Table 2d), CR-DGE (Table 2e), CR-CL (Table 2f), and CR-PPH (Table 2g). The following differences in the DOV after PD were significant: the DOV on POD 3 in the patients with major complications was lower than in those without major complications (174 ml vs. 250 ml, p = 0.008); both the DOVs on POD 1 and POD 3 in patients with IAI were lower than in those without IAI (respectively: 240 ml vs. 286 ml, p = 0.009; 328 ml vs. 410 ml, p < 0.0001); both the DOVs on POD 1 and POD 3 in patients with CR-POPF were lower than in those without CR-POPF (respectively: 178 ml vs. 290 ml, p < 0.0001; 178 ml vs. 250 ml, p = 0.029); both the DOVs on POD 1 and POD 3 in patients with CR-CL were higher than in those without CR-CL (respectively: 485 ml vs. 250 ml, p < 0.0001; 459.5 ml vs. 229.5 ml, p < 0.0001); and the DOV on POD 1 in patients with CR-PPH was higher than in those without CR-PPH (187 ml vs. 275.5 ml, p = 0.027). Figure 1 shows the correlation between the DOV on POD 3 and the serum total protein on POD 3 (Fig. 1a) and serum albumin on POD 3 (Fig. 1b). A significant negative correlation was found for both factors (r = − 0.286, 95% CI – 0.374 to – 0.192, p < 0.001, Fig. 1a), (r = − 0.213, 95% CI – 0.305 to – 0.117, p < 0.001, Fig. 1b).

**Table 2:** Results of a univariate analysis to identify predictors for each complication

	Positive	Negative	p-value
*a. Overall morbidity*			
	209 (51.7%)	195 (48.3%)	
DOV on POD1 (ml)	280 (15–1490)	258 (50–1475)	0.556
DA on POD1 (U/l)	1433 (0–95,100)	429 (9–15,280)	< 0.0001
WBC counts on POD1 (/μl)	10,640 (820–113,800)	10,330 (635–28,350)	0.383
CRP on POD1 (mg/dl)	10.330 (0.79–25.84)	8.970 (0.09–17.50)	< 0.001
DOV on POD3 (ml)	256 (0–1061)	210 (0–1573)	0.085
DA on POD3 (U/l)	163 (3–17,200)	67 (6–11,863)	< 0.0001
WBC counts on POD3 (/μl)	9060 (730–23,710)	8325 (2260–26,230)	0.012
CRP on POD3 (mg/dl)	14.330 (0.87–42.32)	10.470 (0.98–37.57)	< 0.0001
*b. Major complications*			
	100 (24.8%)	304 (75.2%)	
DOV on POD1 (ml)	274.5 (15–1490)	235 (50–1350)	0.165
DA on POD1 (U/l)	1836 (0–95,100)	458 (6–54,600)	< 0.0001
WBC counts on POD1 (/μl)	10,380 (1140–113,800)	10,400 (635–28,350)	0.909
CRP on POD1 (mg/dl)	10.680 (1.42–25.84)	9.130 (0.09–18.40)	< 0.001
DOV on POD3 (ml)	174 (0–1210)	250 (0–1573)	0.008
DA on POD3 (U/l)	213.5 (3–14,272)	72 (5–17,200)	< 0.0001
WBC counts on POD3 (/μl)	10,275 (1460–21,570)	8300 (730–26,230)	< 0.001
CRP on POD3 (mg/dl)	18.890 (3.76–42.32)	10.810 (0.87–37.57)	< 0.0001
*c. IAI*			
	135 (33.4%)	269 (66.7%)	
DOV on POD1 (ml)	240 (50–1475)	286 (15–1490)	0.009
DA on POD1 (U/l)	1836 (0–95,100)	338 (6–54,600)	< 0.0001
WBC counts on POD1 (/μl)	10,840 (1140–113,800)	10,050 (635–28,350)	0.059
CRP on POD1 (mg/dl)	10.440 (0.79–25.84)	9.000 (0.09–17.50)	< 0.001
DOV on POD3 (ml)	328 (0–1210)	410 (0–1573)	< 0.001
DA on POD3 (U/l)	224 (3–17,200)	67 (5–11,863)	< 0.0001
WBC counts on POD3 (/μl)	10,180 (730–23,710)	8275 (2260–26,230)	< 0.0001
CRP on POD3 (mg/dl)	16.350 (3.65–42.32)	10.430 (0.87–37.57)	< 0.0001
*d. CR-POPF*			
	75 (18.6%)	329 (81.4%)	
DOV on POD1 (ml)	178 (50–730)	290 (15–1490)	< 0.0001
DA on POD1 (U/l)	4374 (4–95,100)	458.5 (0–54,600)	< 0.0001
WBC counts on POD1 (/μl)	10,640 (1140–113,800)	10,330 (635–28,350)	0.383
CRP on POD1 (mg/dl)	10.490 (1.26–19.62)	9.280 (0.09–25.84)	0.007
DOV on POD3 (ml)	178 (0–1573)	250 (0–1061)	0.029
DA on POD3 (U/l)	453.5 (3–17,200)	72 (5–11,863)	< 0.0001
WBC counts on POD3 (/μl)	10,420 (1460–21,570)	8510 (730–26,230)	0.005
CRP on POD3 (mg/dl)	18.625 (2.57–42.32)	11.260 (0.87–37.57)	< 0.0001
*e. CR-DGE*			
	23 (5.7%)	381 (94.3%)	
DOV on POD1 (ml)	266 (15–1490)	266 (82–1300)	0.546
DA on POD1 (U/l)	213 (6–16,420)	735 (0–95,100)	0.092
WBC counts on POD1 (/μl)	10,400 (635–113,800)	9390 (5300–17,460)	0.28
CRP on POD1 (mg/dl)	9.245 (5.01–15.58)	9.495 (0.09–25.84)	0.7
DOV on POD3 (ml)	236 (0–1573)	212 (35–830)	0.693
DA on POD3 (U/l)	46 (9–1289)	104 (3–17,200)	0.237
WBC counts on POD3 (/μl)	7710 (2630–13,220)	8740 (730–26,230)	0.161
CRP on POD3 (mg/dl)	11.370 (3.37–29.36)	12.365 (0.87–42.32)	0.639
*f. CR-CL*			
	20 (5.0%)	384 (95.0%)	
DOV on POD1 (ml)	485 (144–1332)	250 (15–1490)	< 0.0001
DA on POD1 (U/l)	978 (4–15,944)	705.5 (0–95,100)	0.928
WBC counts on POD1 (/μl)	9620 (820–17,090)	10,400 (635–113,800)	0.464
CRP on POD1 (mg/dl)	9.595 (3.53–15.37)	9.495 (0.09–25.84)	0.945
DOV on POD3 (ml)	459.5 (130–1573)	229.5 (0–1210)	< 0.0001
DA on POD3 (U/l)	119 (3–2811)	100 (5–17,200)	0.787
WBC counts on POD3 (/μl)	8475 (3150–17,030)	8640 (730–26,230)	0.526
CRP on POD3 (mg/dl)	7.730 (1.10–21.45)	12.445 (0.87–42.32)	0.004
*g. CR-PPH*			
	28 (6.9%)	376 (93.1%)	
DOV on POD1 (ml)	187 (60–867)	275.5 (15–1490)	0.027
DA on POD1 (U/l)	2313 (63–38,492)	639 (0–95,100)	< 0.0001
WBC counts on POD1 (/μl)	10,590 (1140–19,190)	10,380 (635–113,800)	0.919
CRP on POD1 (mg/dl)	10.705 (6.54–16.02)	9.465 (0.09–25.84)	0.189
DOV on POD3 (ml)	195 (30–980)	240 (0–1573)	0.165
DA on POD3 (U/l)	217.5 (43–13,230)	91.5 (3–17,200)	< 0.0001
WBC counts on POD3 (/μl)	11,175 (1460–21,570)	8560 (730–26,230)	0.033
CRP on POD3 (mg/dl)	21.090 (4.57–42.32)	11.370 (0.87–37.57)	< 0.0001

*DOV* drain output volume, *DA* drain amylase, *WBC* white blood cell, *CRP* c-reactive protein, *POD* postoperative day, *IAI* intraabdominal infection, *CR* clinically relevant, *POPF* postoperative pancreatic fistula, *DGE* delayed gastric emptying, *CL* chyle leak, *PPH* post-pancreatectomy hemorrhage

### Predictability of the DOV for postoperative complications

The development of major complications, IAI, CR-POPF, CR-CL and CR-PPH was shown to be correlated with the DOV on POD 1 and/or POD 3, so we further evaluated the independent postoperative warning sign for these complications by multivariate analyses. Table 3a shows that a higher CRP on POD 1 (OR 2.45, 95% CI: 1.1–5.47, p = 0.028), higher DA on POD 3 (OR 2.89, 95% CI: 1.46–5.73, p = 0.002) and higher CRP on POD 3 (OR 2.25, 95% CI: 1.38–7.68, p = 0.007) were independent postoperative warning signs of the development of major complication. Table 3b shows that a higher DA on POD 1 (OR 3.59, 95% CI: 1.77–3.13, p < 0.001), higher CRP on POD 1 (OR 1.77, 95% CI: 1–3.13, p = 0.048), and higher CRP on POD 3 (OR 2.85, 95% CI: 1.65–4.9, p < 0.001) were independent postoperative warning signs of the development of major complications. Table 3c shows that a lower DOV on POD 1 (OR 3.88, 95% CI: 1.89–4.35, p < 0.001), higher DA on POD 1 (OR 6.8, 95% CI: 3.24–14.3, p < 0.0001), higher DA on POD 3 (OR 3.56, 95% CI: 1.4–9.09, p = 0.008) and higher CRP on POD 3 (OR 3.41, 95% CI: 1.68–6.95, p < 0.001) were independent postoperative warning signs of the development of CR-POPF. Table 3d shows that a higher DOV on POD 3 (OR 5.36, 95% CI: 1.56–18.4, p = 0.008) was an independent postoperative warning sign of the development of CR-CL. Table 3e shows that a higher CRP on POD 3 (OR 11.6, 95% CI: 2.16–51.5, p = 0.001) was an independent postoperative warning sign of the development of CR-PPH.

**Table 3:** Results of a multivariate analysis to identify predictors for each complication correlated with the drain output volume

	Odds ratio	95% CI	p-value
*a. Major morbidity*			
DA on POD1 ≥ 913 (U/l)	2.89	0.573–2.51	0.63
CRP on POD1 ≥ 10.09 (mg/dl)	2.45	1.1–5.47	0.028
DOV on POD3 ≥ 130 (ml)	0.734	0.323–1.67	0.461
DA on POD3 ≥ 96 (U/l)	2.89	1.46–5.73	0.002
WBC counts on POD3 ≥ 10,370 (/μl)	1.38	0.605–3.160	0.443
CRP on POD3 ≥ 13.4 (mg/dl)	3.25	1.38–7.68	0.007
*b. IAI*			
DOV on POD1 ≥ 380 (ml)	0.602	0.328–1.110	0.102
DA on POD1 ≥ 608 (U/l)	3.59	1.77–7.290	< 0.001
CRP on POD1 ≥ 8.58 (mg/dl)	1.77	1–3.13	0.048
DOV on POD3 ≥ 125 (ml)	0.609	0.338–1.1	0.099
DA on POD3 ≥ 96 (U/l)	1.38	0.692–2.73	0.363
WBC counts on POD3 ≥ 10,560 (/μl)	1.46	0.8330–2.55	0.187
CRP on POD3 ≥ 13.95 (mg/dl)	2.85	1.65–4.9	< 0.001
*c. CR-POPF*			
DOV on POD1 ≥ 227 (ml)	0.258	0.123–0.529	< 0.001
DA on POD1 ≥ 2180 (U/l)	6.8	3.24–14.3	< 0.0001
CRP on POD1 ≥ 7.82 (mg/dl)	2.04	0.822–5.06	0.124
DOV on POD3 ≥ 190 (ml)	1.32	0.646–2.71	0.443
DA on POD3 ≥ 103 (U/l)	3.56	1.4–9.09	0.008
WBC counts on POD3 ≥ 11,160 (/μl)	1.59	0.769–3.27	0.211
CRP on POD3 ≥ 13.94 (mg/dl)	3.41	1.68–6.95	< 0.001
*d. CR-CL*			
DOV on POD1 ≥ 390 (ml)	2.85	0.899–9.01	0.075
DOV on POD3 ≥ 332 (ml)	5.36	1.56–18.4	0.008
CRP on POD3 ≥ 13.07 (mg/dl)	0.361	0.0988–1.32	0.123
*e. CR-PPH*			
DOV on POD1 ≥ 206 (ml)	0.432	0.186–1.00	0.051
DA on POD1 ≥ 457 (U/l)	1.25	0.29–5.43	0.763
DA on POD3 ≥ 55 (U/l)	6.01	0.59–61.2	0.13
WBC counts on POD3 ≥ 11,160 (/μl)	2	0.85–4.67	0.111
CRP on POD3 ≥ 13.55 (mg/dl)	11.6	2.61–51.6	0.001

*DOV* drain output volume, *DA* drain amylase, *WBC* white blood cell, *CRP* c-reactive protein, *POD* postoperative day, *IAI* intraabdominal infection, *CR* clinically relevant, *POPF* postoperative pancreatic fistula, *CL* chyle leak, *PPH* post-pancreatectomy hemorrhage

### Diagnostic accuracy of drain output volume for CR-POPF and CR-CL

**Fig. 2:**
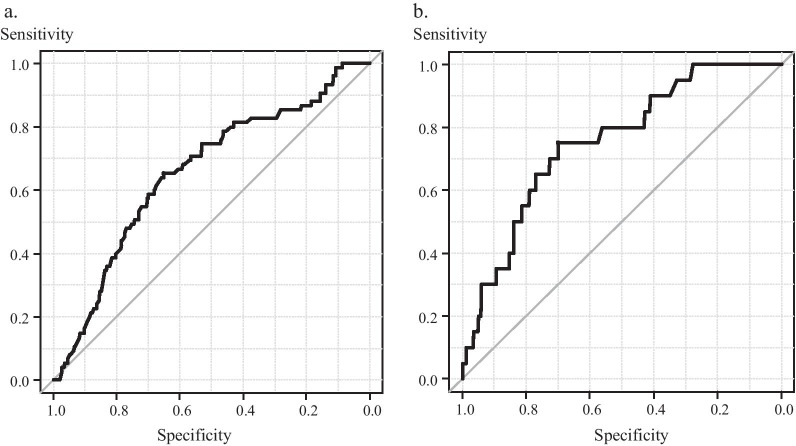
Receiver operating characteristics (ROC) curves of drain output volume (DOV) to determine the cutoff value of DOV on POD 1 to predict CR-POPF (**a**), and DOV on POD 3 to predict CR-CL (**b**) after pancreaticocuodenectomy. ROC curve (thick line), 95% confidence interval (CI) bounds (narrow line)

A low DOV on POD 1 was an independent postoperative warning sign of CR-POPF, and a high DOV on POD 3 was an independent postoperative warning sign of CR-CL. An ROC analysis revealed that the DOV on POD 1 had a negative predictive value (area under the curve (AUC) 0.655, sensitivity 65.0%, specificity 65.3%, 95% CI: 0.587–0.724), with a calculated optimal cut-off value of 227 ml (Fig. 2a). An ROC analysis also revealed that the DOV on POD 3 had a positive predictive value (AUC 0.753, sensitivity 70.1%, specificity 75.0%, 95% CI: 0.651–0.856), with a calculated optimal cut-off value of 332 ml (Fig. 2b).

## Discussion

Many studies have evaluated the preoperative risk factors and postoperative warning signs of CR-POPF after PD. Absence of main pancreatic duct dilatation and normal texture of pancreas were well known as preoperative and intraoperative risk factors for CR-POPF after PD [19–21]. High DA and/or high CRP on POD 1 and/or POD 3 were strong postoperative warning signs of CR-POPF after PD [1, 4–7]. In 2 recent retrospective series, a DA on POD 1 ≥ 303 U/l and CRP on POD 3 > 20.3 mg/dl [7] and a DA on POD 3 ≥ 350 U/l and CRP on POD 3 ≥ 14 mg/dl [6] were able to accurately predict CR-POPF. High DA on POD3 was a strong postoperative warning sign for CR-POPF [3]; however, it was also found in patients who did not develop CR-POPF in this series. POPF does not progress to CR–POPF in all cases, and the development of CR–POPF is frequently triggered by infection in patients with POPF [3, 6–9]. High CRP on POD 3 might reflect systemic inflammation and concomitant infection [6–9]. In this series, we also found that high DA on POD 1 (≥ 2180 U/l) and POD 3 (≥ 103 U/l), as well as high CRP on POD 3 (≥ 13.9 mg/dl), were independent predictors of CR-POPF. These findings underscore the utility of the combination of DA and CRP for the prediction of CR-POPF.

In addition to the above, we newly showed that a low DOV on POD 1 (< 227 ml) was a potential postoperative warning sign of CR-POPF. Ito et al. reported that the CR-POPF was significantly lower in patients with a high DOV (DOV > 10 ml/kg/day) than in those with a low DOV (DOV ≤ 10 ml/kg/day) after PD for pancreatic ductal adenocarcinoma, but the cut-off value was not evaluated [14]. An increased DOV dilutes the amylase concentration in the drainage output, which reduces the development of POPF and the consequent infection rate [14, 22]. Alternatively, in patients with a low DOV, the low amount of intra-abdominal ascites may fail to dilute the amylase level, which might increase the incidence of POPF. Another possible reason for the predictability of a low DOV for CR-POPF is the dislocation and dysfunction of drains. The dislocation and dysfunction of drains are early and frequent events after PD [23]. In such situations, the DOV may be reduced, and the drain itself may act as a foreign body, causing an inflammatory response and infection. Undrained peripancreatic fluid collection during the early postoperative period is frequent and is associated with the development of CR-POPF [24]. We do not routinely conduct computed tomography during the early postoperative period in our institution, so we were unable to evaluate the undrained fluid collection in most cases in this retrospective series. Therefore, unfortunately, we cannot further discuss the correlations among the drain dysfunction, DOV and development of CR-POPF. A low DOV on POD 1 may be a postoperative warning sign for CR-POPF, similar to the presence of a high DA and high CRP on POD 1 and/or POD 3. When the DOV on POD 1 is low, surgeons should be alert for the development of CR-POPF and therefore try to determine why the DOV is low. The predictive power of a low DOV on POD 1 for CR-POPF was significant but low, so further large-scale studies will be necessary to validate this correlation and the cut-off value of DOV for predicting CR-POPF.

Preoperative risk factors and postoperative warning signs for CL after pancreatic resection have been evaluated; however, they are highly heterogeneous [11]. Early enteral feeding, manipulation of the para-aortic area and the extent of LN dissection or total number of harvested LNs have been reported as risk factors for CL [11, 25–27]. The postoperative warning signs of CR-CL before its development has been difficult and not studied well. Kim et al. reported that a DOV of > 335 ml on POD 4 indicated suspected CL [22], which was consistent with the present findings. A recent study by Shyr et al. showed that the DOVs from POD 1–7 were significantly higher in patients with CL than in those without CL, but unfortunately, they did not evaluate the cut-off value to predict CL [27]. In the present study, we showed that a DOV on POD 3 ≥ 332 ml was an independent predictor of the development of CR-CL following PD. CL usually is recognized by the appearance of milky/white drainage fluid with the start of oral intake at a median of POD 5 to POD 6 [11]. The early detection of CR-CL helps shorten the postoperative hospital stay and reduce medical costs, as conservative treatment is effective [11, 28]. In addition, as in previous studies [13, 14], we also showed that a high DOV was associated with significant protein loss. This protein loss causes a decrease in the plasma osmolality and may increase transudative ascites, creating a vicious cycle that may increase the DOV. Therefore, patients with a high DOV on POD 3 should be monitored for CR-CL and might require careful management of their massive protein loss.

Several limitations associated with the present study warrant mention. First, this was a retrospective study, and the study cases were collected over a long period. There may have been some variance in the data, including the operation technique and timing of drain removal. While a long duration of drain placement is known to cause infectious complications, a high DOV or changing vital signs may have prompted surgeons to keep drains in place for a long period of time. Second, our sample size was limited. The frequencies of overall mortality and morbidity, major complications and CR post-pancreatectomy complications, including CR-POPF, CR-DGE, CR-CL and CR-PPH, were comparable to those in previous reports from high-volume hospitals [5, 12–14]. However, the limited number of samples may have weakened the statistical power of this study.

## Conclusion

A low DOV on POD 1 might be a postoperative warning sign for CR-POPF, similar to high DA on POD 1, high DA on POD 3, and high CRP on POD 3. When the DOV on POD 1 after PD is low, surgeons should evaluate the reasons for the low DOV. A high DOV on POD 3 was shown to be a postoperative warning sign for CR-CL and might require careful management of protein loss. Further large-scale studies are necessary to validate these findings and the cut-off value of DOV for predicting CR-POPF and CR-CL.

## Data Availability

The datasets used and/or analysed during the current study are available from the corresponding author on reasonable request.

## Abbreviations

PD: Pancreaticoduodenectomy
ISGPS: The International Study Group of Pancreatic Surgery
POPF: Postoperative pancreatic fistula
DA: Drain amylase
POD: Postoperative day
CR: Clinically relevant
DOV: Drain output volume
DGE: Delayed gastric emptying
CL: Chyle leak
PPH: Post-pancreatectomy hemorrhage
WBC: White blood cell
CRP: C-reactive protein
OR: Odds ratio
CI: Confidence interval
ROC: Receiver operative characteristic
AUC: Area under the curve
